# Platelet-Rich Plasma With an Activator: A New Approach for Treating Thin Endometriums of Women Suffering From Infertility

**DOI:** 10.7759/cureus.70495

**Published:** 2024-09-30

**Authors:** Neha Nawale, Akash More, Namrata Choudhary, Jarul Shrivastava, Sanket Mahajan

**Affiliations:** 1 Clinical Embryology, Datta Meghe Institute of Higher Education and Research, Wardha, IND

**Keywords:** female infertility, introcytoplasmic sperm injection (icsi), in vitro fertilization ivf, platelet-rich plasma (prp), prp activation

## Abstract

This case study reflects the primary infertility treatment which was in vitro fertilization (IVF) that lasted for four years. This case study involves a 37-year-old female who underwent intrauterine insemination (IUI), recurrent implantation failure, and frozen embryo transfers with a focus on platelet-rich plasma (PRP) for receptor endometrial thickness. The patient's report revealed a hormonal imbalance and a thin endometrium measuring 5 mm, while her husband's semen analysis findings were normal. Activation with calcium chloride PRP therapy successfully helped to achieve an endometrial thickness of 7.1 millimeters, and then, on the second day of the menstrual cycle, gonadotropin-releasing hormone (GnRH) was given. Six oocytes were retrieved, and after intracytoplasmic sperm injection (ICSI), a good-quality blastocyst was developed. On day 21 of the cycle, an embryo of 4AB grade was transferred. Serum beta-human chorionic gonadotropin (β-hCG) was positive, which confirmed pregnancy 14 days after the process of embryo transfer. The patient was advised to take regular check-ups during pregnancy. Recurrent implantation failure and thin endometrium are critical issues in this case, demonstrating that PRP can be used to augment the endometrial thickness to improve the pregnancy rate.

## Introduction

According to the World Health Organization (WHO), infertility is ‘‘a pathological condition of the reproductive system that prevents pregnancy after 12 months of regular sexual intercourse'' [1]. During reproduction, the endometrium plays a crucial role, which can be observed through grayscale and 3D ultrasound imaging. In assisted reproductive technology (ART), endometrial receptivity has been crucial in maintaining pregnancy with successful outcomes [2]. Endometrial thickness is measured via transvaginal ultrasound by identifying the echoes from the interfaces between the myometrium and endometrium in a plane that was largely horizontal to the longitudinal axis of the fundus. Endometrial thickness of less than 7 mm was formerly considered suboptimal for embryo transfer (ET) [3]. A thin endometrium has led to poor implantation, infertility, and the ending of ART treatments. Under these conditions, there had been increased incidences of premature deliveries, babies with low birth weights, and early fetal loss [4].

With recent advancements in regenerative medicine, platelet-rich plasma (PRP) has been used in orthopedics, cardiothoracic surgery, plastic surgery, dermatology, dentistry, and diabetic wound healing. However, the majority of this research indicates that PRP is effective; however, there isn't much conclusive evidence to support its practitioners [5]. Platelet-rich plasma, a concentrated blood product with high platelet content, has been used in cases with recurrent implantation failure. Whole blood contains red and white blood cells, blood platelets, and plasma. The biggest part of the blood composition was plasma, which included water, proteins, and dissolved ions, and this part constituted half of the total blood quantity. Plasma has also served as a transport medium for all the other portions of the body [6]. Platelet-rich plasma is created using freshly drawn whole blood from a patient and produced by centrifuging red blood cells, which contain mending and anti-inflammatory qualities [7].

Certain approaches, such as fibrin, thrombin, or calcium chloride, have activated PRP. It had been demonstrated that stimulating platelets before treatment would boost the release of the correct growth factors, increasing the therapeutic efficacy of platelet-derived PRP. Research has shown that the activated platelet-derived growth factor exhibited improved results compared to the non-activated PRP; hence, the activation of PRP was required for the most benefits in the treatment [8]. Platelet activation had been caused by various agonists, including subendothelial collagens, thromboxane A2 (TxA2), adenosine diphosphate (ADP) released by activated platelets, and thrombin produced by the coagulation cascade. Although these agonists had acted on various platelet receptors and initiated different signaling pathways, they had all increased intracellular calcium ion (Ca2+) concentration [9].

## Case presentation

Patient information

This study revolved around a couple who had experienced four years of primary infertility. They visited our in vitro fertilization (IVF) center in Nagpur, Maharashtra, India. The male was 40 years old, and the female was 37 years old. They were given a full description of the benefits and drawbacks of the procedures. Informed consent was obtained from both individuals.

Medical history

The patient had undergone four intrauterine inseminations (IUI) and two failed frozen embryo implantation cycles. Neither partner had a medical history of heart problems, tuberculosis (TB), or hypertension. This had been their first time receiving infertility treatment at our center.

Physical examination

The female's body mass index (BMI) was 22.9 kg/m², and the male's was 27.3 kg/m². The physical examination revealed that all patterns had been within the normal range.

Investigation

Based on the husband's semen report, the count was 98 million/mL, semen morphology was 96%, motility was 90%, and the normal morphology of the semen was 4%. The investigation had determined that the report was normal. Table 1 shows the husband's semen analysis report.

**Table 1:** Semen anaylsis report of the patient's husband *The sixth edition of the WHO Manual for the Laboratory Examination and Processing of Human Semen [10]

Parameter	Observed limit	Reference limit (WHO 2020)*
Semen volume	1.6 mL	>1.4 mL
Morphological defects	96%	96%
Normal morphology	4%	>4 %
Vitality	48%	>54%
Progressive motility	36%	>30%
Count	98 million/mL	16 million/mL
pH	7.0	>7.2
Color	Opaque white	Opaque white
Viscosity	Liquified	

The ultrasonography report of the patient revealed a thin endometrium measuring 5.8 mm. Her hormone levels were abnormal: anti-Müllerian hormone (AMH) had been 0.81 ng/ml, and follicle-stimulating hormone (FSH) was 22 IU/L, as shown in Table 2.

**Table 2:** Hormonal levels of the patient Source: [11]

Hormonal profile	Patient values	Reference values
Anti-Müllerian hormone	0.81 ng/ml	0.8-1.0 ng/ml
Follicle-stimulating hormone	22 IU/L	10 IU/L

Treatment

**Figure 1:**
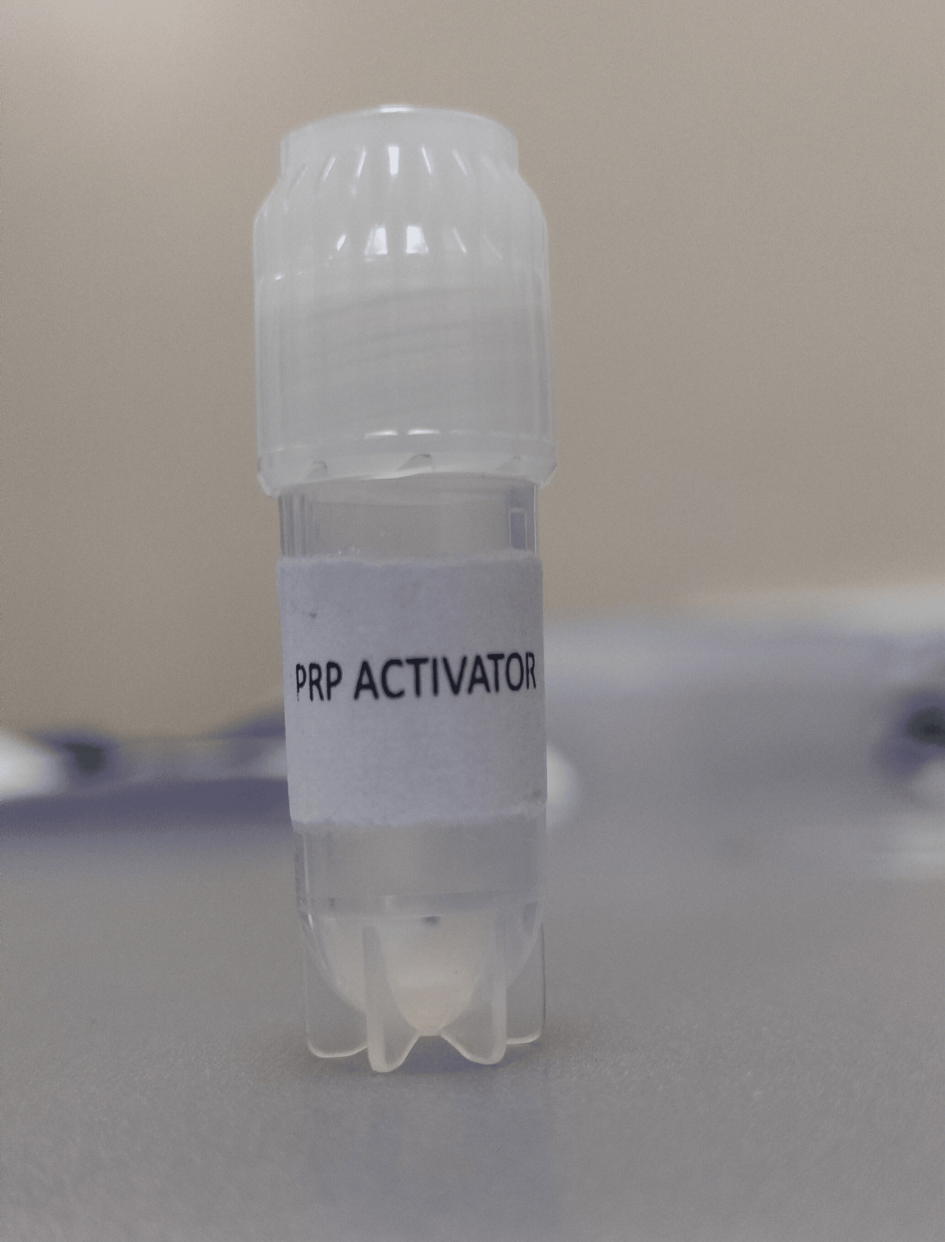
The PRP activator used to increase endometrial thickness PRP: platelet-rich plasma

Due to her thin endometrium, the patient had been recommended to receive PRP treatment. Three days later, the ultrasonographic result showed 5.8 mm of endometrial thickness. After one month, the patient's thin endometrium was still deemed to warrant PRP therapy. We added 1.5 mL of anticoagulant to the two 15 mL test tubes; 13.5 milliliters of blood were drawn using scalp vein set 18-nine anticoagulant. The tube caps had been turned upside down several times to ensure they were securely closed and well mixed. Then, the tubes were centrifuged at 1,000 revolutions per minute (RPM) for 10 minutes. Following centrifugation, we isolated PRP, ensuring the red blood cell (RBC) count had at least 50% plasma volume without the buffy coat. Next, we used a sterile pipette to transfer the plasma to a different test tube without removing the RBCs. After that, the plasma was collected in a tube. To obtain a platelet pellet, we spun the centrifuge a second time for 10 minutes at 2,000 RPM. The ensuing upper plasma layer was evident. The leftover plasma was removed using a sterile pipette, leaving 1 mL on top of the pellet. Then, 10% calcium chloride was added to activate the PRP, as shown in Figure 1. 

**Figure 2:**
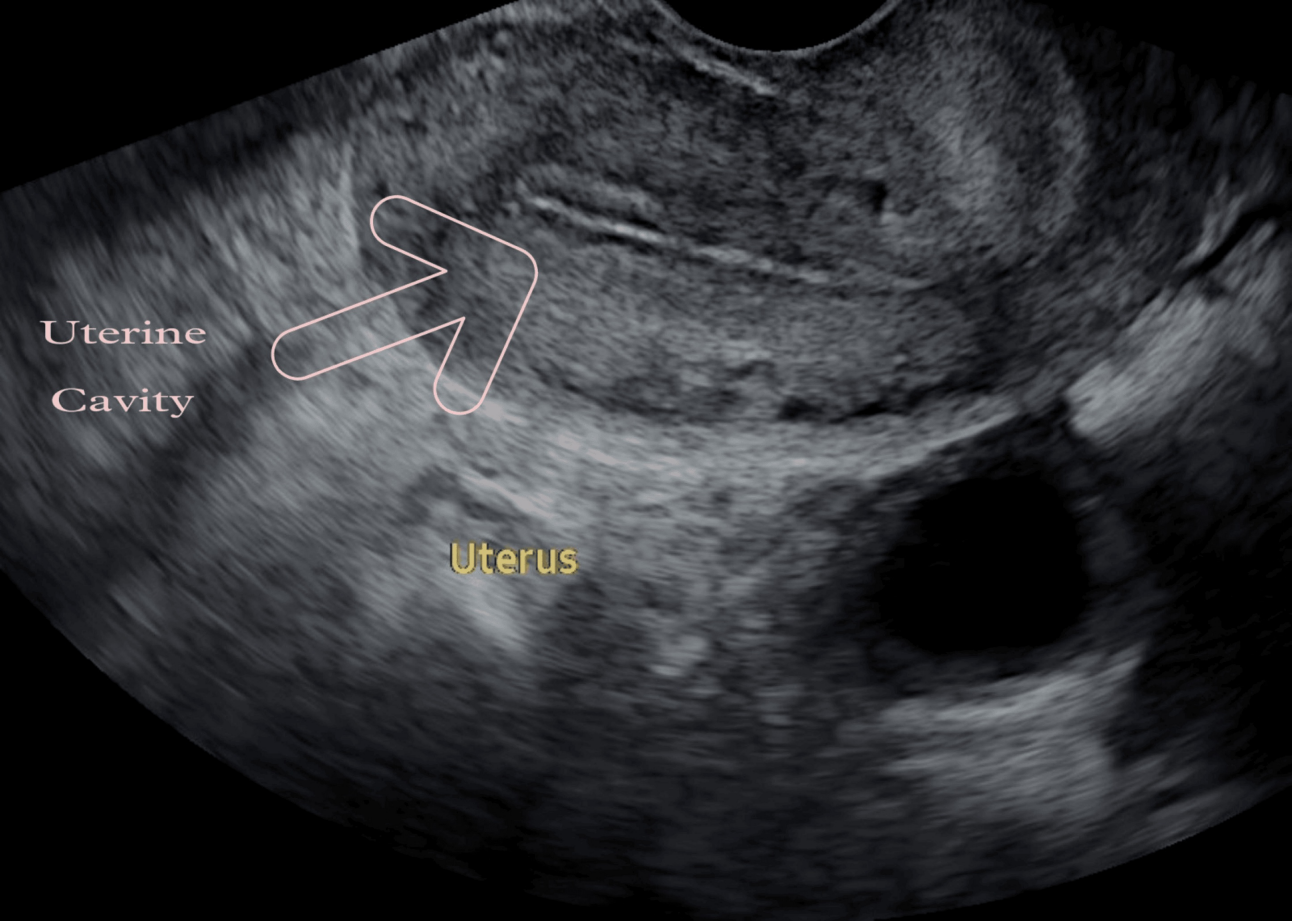
Endometrial thickness of 7.1 mm is noted

The activated PRP was collected in a syringe while maintaining sterilization. A soft catheter was used to infuse 0.5 to 1 mL of activated PRP into the uterus. After 72 hours, an ultrasonography report revealed an endometrial thickness of 7.1 mm, as shown in Figure 2.

A gonadotrophin-releasing hormone (GnRH) agonist had been suggested as the treatment for the patient on day two of her menstrual cycle. On day 14, we recommended a subcutaneous injection of 10,000 IU of human chorionic gonadotropin (hCG) for the maturation of the oocytes. After 36 hours of being triggered, the patient was recommended for oocyte pickup. Six oocytes (one metaphase II (MII), three metaphase I (MI), and two germinal vesicles (GV)) had been retrieved during the process. On the same day, intracytoplasmic sperm injection (ICSI) was administered. By day five, a good-quality blastocyst had formed. On day 21 of the menstrual cycle, the patient was advised to undergo ET, where an embryo of 4AB grade was transferred.

Follow-up

After the ET, the patient was instructed to start taking her regular medication regularly. After 14 days, her blood sample's serum beta-hCG level was determined to be 1,586 mIU/mL. After conception, we advised the patient to come to the clinic often.

## Discussion

Zadehmodarres et al. demonstrated the first evidence that endometrial growth in women with thin endometrium could be stimulated by intrauterine PRP injection. During the frozen embryo transfer cycle, PRP was administered to five women who had insufficient endometrium thickness and had not responded well to traditional therapy [12]. We conducted this investigation on a single lady to increase endometrial thickness during frozen embryo transfer. According to Soliman et al., intrauterine infusion of PRP could boost implantation and pregnancy outcomes in recurrent implantation failure (RIF) patients; thus, clinicians should consider this intervention a constructive approach in assisted reproductive procedures [13]. In our case report, we infused intrauterine PRP in a patient with a thin endometrium. Endometrial thickness is the most crucial factor in IVF, followed by a high-quality embryo [14]. When following a preparatory protocol, fresh and frozen embryo transfers could achieve the required endometrial thickness of over 7 mm. Certain patients whose live birth rate was lower but still acceptable should have been recommended to move forward with the embryo transfer even if they could not acquire an endometrial thickness in many preparation cycles [4].

This chemical causes platelets to degrade and release their growth factors by triggering a rapid coagulation response in PRP calcium chloride activation, which is cheap, fast, and readily available. [15] The present novel method proved particularly effective for clinical uses where direct and easy activation methods were needed. Other substances that activate platelets include ADP, thrombin, epinephrine, thromboxane A2, collagen, and many others. This activation caused the aggregation of platelets through the interaction between the fibrinogen and glycoprotein IIb/IIIa receptors. Also, it enhanced the density of other surface molecules referred to as ‘activation markers’ on the activated platelets, including CD62P and CD63. Morphological characteristics of the platelets have evolved, and the various shapes include discoid, non-rolling ball-like, hemispherical, and spreading adhesion-like forms [16].

## Conclusions

Platelet-rich plasma has been used and applied to the following case reports as a PRP indicator. The patient's history of primary infertility with recurrent implantation failure also involved one or two IUI attempts and frozen embryo transfers. In the patient’s report, a thin endometrium had been documented, which was an indication of poor implantation and infertility. Patients who received a treatment of PRP with a PRP activator were undergoing endometrial thickness augmentation from 5.8 to 7.2 mm. The enhancement made it possible to formulate more quality blastocysts, which had previously been transplanted during ART. The patient’s β-hCG level pointed out positive results showing that the patient was pregnant.
